# Multidetector computed tomography of chest trauma: indications, technique and interpretation

**DOI:** 10.1007/s13244-012-0187-7

**Published:** 2012-08-04

**Authors:** Hynek Mirka, Jiri Ferda, Jan Baxa

**Affiliations:** Department of Imaging methods, Charles University and University Hospital in Pilsen, Alej Svobody 80, 304 60 Pilsen, Czech Republic

**Keywords:** Multidetector computed tomography, Thorax, Wounds and injuries, Blunt trauma, Penetrating trauma

## Abstract

**Background:**

Chest traumas are a significant cause of mortality and morbidity, especially in the younger population.*Methods*Diagnostic imaging plays a key role in their management. Multidetector computed tomography (MDCT) is the most important imaging method in this field. Its advantages include especially high speed and high geometric resolution in any plane.*Results*The method allows us to view large parts of the body with minimal motion artifacts and to create accurate multiplanar and three-dimensional (3D) reformations, which make the diagnosis significantly more accurate. Because of its advantages MDCT has become the first-choice method in high-energy traumas.*Conclusion*This article summarises the position of MDCT in the diagnostic algorithm of chest injuries, technical aspects of the examination and imaging findings in traumas of the individual chest compartments.

**Teaching Points:**

• *Diagnostic imaging plays a key role in the management of high-energy chest trauma.*

• *MDCT is the most important imaging method in this kind of injury, as detailed information can be acquired in a short acquisition time.*

• *Multiplanar and three-dimensional (3D) reformattings make the diagnosis significantly more accurate.*

## Introduction

In industrialised countries injuries represent a significant socioeconomic problem. They commonly occur in the younger population and are the most common cause of death in people between 25 and 44 years of age. Chest injuries occur in about 20 % of all trauma patients. In up to 80 % of cases they are associated with injuries of other body parts such as the head (69 %), abdomen and pelvis (43 %) and extremities (52 %) [1, 2]. Diagnostic imaging plays a key role in deciding on the therapeutic procedure and determining the prognosis [3]. Multidetector computed tomography (MDCT) is considered to be the most effective imaging method in this field and therefore should be an integral part of the emergency department [4]. This article summarises the position of MDCT in the diagnostic algorithm, examination technique and findings in injuries of the individual chest compartments.

## Mechanisms of injury, classification

**Fig. 1:**
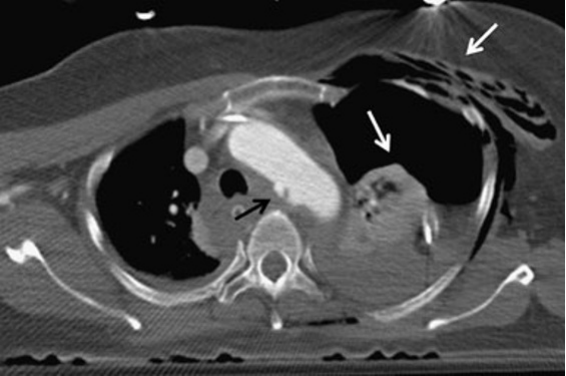
Combination of two traumatic mechanisms, pedestrian knocked down by a car. Rib fractures, hemothorax and pneumothorax on the left side are results of direct impact (*white arrows*). Small aortic pseudoaneurysm is an effect of deceleration (*black arrow*)

Chest injuries usually occur in car crashes, falls from heights, sports injuries and violent acts. The most affected compartments include the chest wall (>50 %), pleura (50 %) and lungs (30–70 %). Rare but often very severe are traumas of the airways (2.8 to 5.4 %), diaphragm (0.4–1.5 %), large vessels (1.1 to 2.2 %) and heart (10 %) [3, 5]. According to the underlying cause, the injuries are classified as blunt (90 %) and penetrating (10 %) injuries. Blunt injury is caused by three basic mechanisms, which are often combined. These are a direct impact, compression and deceleration [4]. Direct impact on the chest causes a localised injury of the chest wall at the point of contact. When exposed to major forces, the energy can affect the deeper structures located in their trajectory, such as lungs, heart, blood vessels, mediastinum, liver or spleen. Compression causes pressing of the organs against the spine or chest wall and their contusion or rupture. The pulmonary parenchyma, pleura, diaphragm and tracheobronchial tree are often affected. Deceleration causes movement of the organs and shearing forces at the site where they are fixated. This generates traumas of the tracheobronchial tree, aorta, heart and diaphragm (Fig. 1). The causes of a penetrating trauma are often stab and gunshot wounds in victims of violence and war. Due to the higher incidence of vascular and heart injuries, a penetrating trauma has a higher mortality compared to a blunt trauma [6].

## Imaging methods

**Fig. 2:**
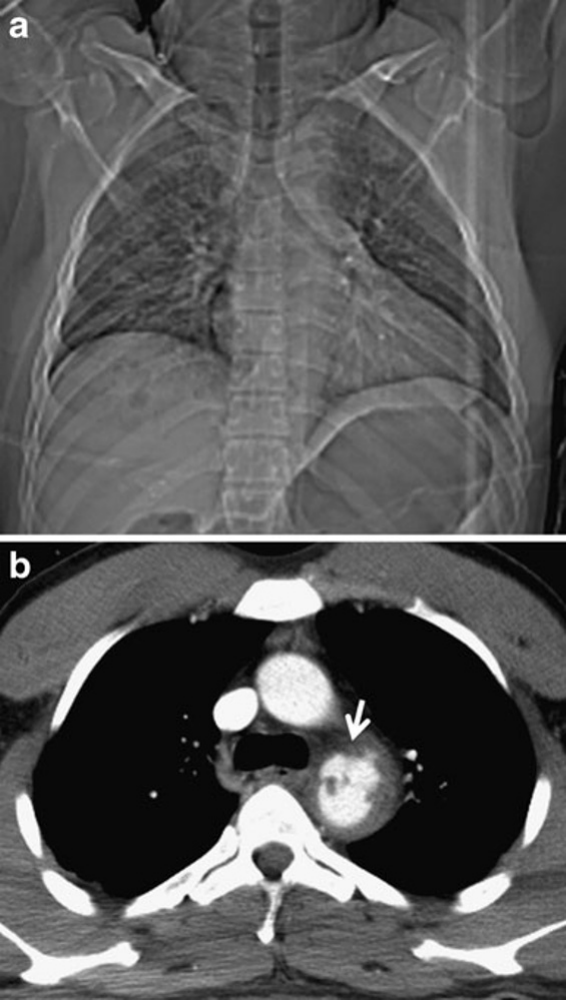
**a, b**Patient with laceration of the aorta. **a**Topogram (analogical to chest radiograph) shows normal mediastinal width. **b**MDCT reveals rupture of the aortic isthmus with small periaortic hematoma (*arrow*)

Clinical symptoms of chest injuries are diverse and often do not correlate with their severity. This is the reason why diagnostic imaging belongs among the first procedures performed after admission to a medical facility. The simplest and fastest feasible methods include chest radiography and ultrasound. They can, especially in unstable patients, provide important information about the presence of serious injuries requiring an emergency intervention, such as tension pneumothorax, large hemothorax, hemopericardium, hemoperitoneum and injuries of the abdominal organs under the diaphragm. These methods are less sensitive for some types of trauma, and therefore, especially in high-energy injuries they cannot be considered as conclusive. Chest radiography is performed in severe injuries in supine position, which further reduces its contribution. Detection of a contusion and laceration of the pulmonary parenchyma, but also a smaller hemothorax and pneumothorax, is also an issue. It is also very unreliable in detecting injuries of the heart and great vessels [7–11]. In addition to low sensitivity in certain types of traumas, its low specificity is a problem. A symptom of expanded mediastinum, which may be a sign of trauma of the major vessels and heart, can be an example. However, this concerns only about 20 % of cases. The remaining findings are caused by bleeding without trauma of the major blood vessels or also by non-traumatic causes such as mediastinal lipomatosis, congenital abnormalities, enlarged lymphatic nodes or tumors [5, 12]. Compared to this, MDCT enables sufficiently accurate assessment of all compartments of the chest and also reveals changes that are not detectable by other methods (Fig. 2). Additional findings are very common in MDCT (up to 83 % of cases), but in fact only some of them can change the therapeutic process (7–41 %) [10, 13–15].

## MDCT

**Fig. 3:**
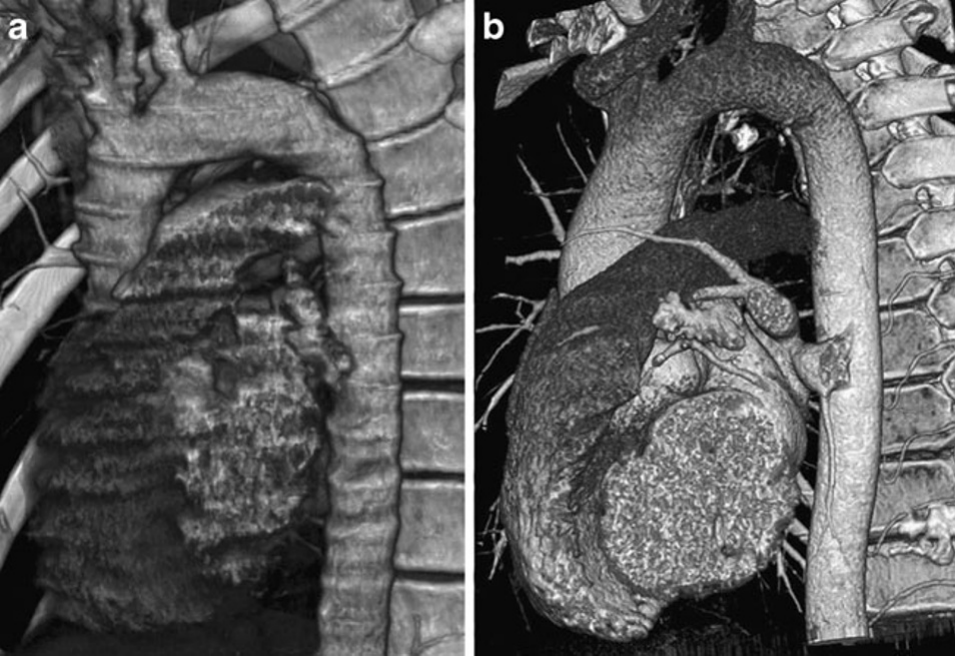
**a, b**Effect of acquisition speed on the quality of 3D images. **a**Six-row MDCT, acquisition time 20 s: significant motion artifacts on the heart and aorta. **b**Sixty-four-row MDCT, acquisition time 7 s: heart and aorta are free of artifacts

Implementation of MDCT has significantly increased the accuracy of diagnosis of injuries, not only in the area of the chest, and is now considered as a fundamental method in trauma radiology. It is able to comprehensively examine all structures of the chest with a sensitivity and specificity approaching 100 %. Excellent spatial and temporal resolution is the main benefit. Because of the isotropic data field, it allows performing two-dimensional (2D) and three-dimensional (3D) reformations in any plane and angle of view without loss of geometric resolution and to evaluate the anatomical structures, which are located adversely to the axial plane. A high-speed examination is needed to view the entire examination area in the proper post-contrast circulation phase and to minimise motion artifacts (Fig. 3) [4, 16, 17].

### Diagnostic algorithm

**Fig. 4:**
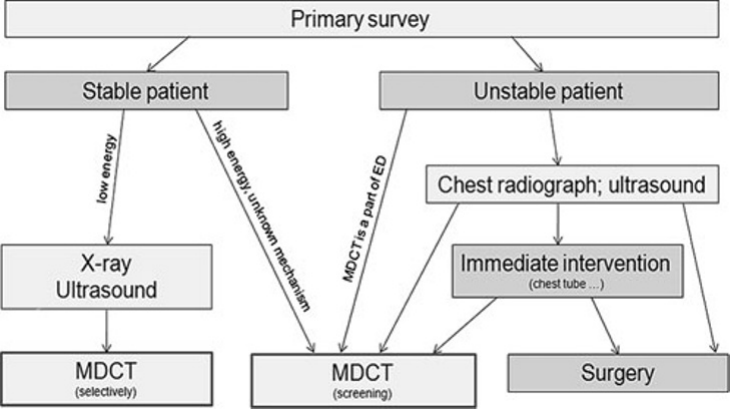
Diagnostic algorithm used in our institution (ED, emergency departement)

The choice of a diagnostic procedure depends on the patient’s condition and traumatic mechanism. In low-energy traumas (falls from heights of up to 3 m and traffic accidents at speeds of up to 50 km/h), the standard method includes X-ray and ultrasound examinations. The chest MDCT examination should be performed only in case of unclear findings or if more detailed assessment is needed. In patients with high-energy trauma (falls from heights above 3 m and traffic accidents at speeds exceeding 50 km/h) and unknown mechanism of trauma, the chest MDCT examination should be performed as a screening method. The examination is usually a part of the whole-body CT. In stable patients with no apparent need of emergency intervention, the MDCT examination can be performed directly after admission to a health-care facility and primary clinical examination. In the case of circulatory instability or if pharmacological circulatory support is needed, the chest X-ray in supine position is usually performed as well as ultrasound examination to exclude findings that require an immediate intervention. The MDCT examination should be performed only after these necessary procedures have been completed [18]. In the event that MDCT is a part of the emergency department and the examination can be performed concurrently with resuscitation, this step can be skipped, and the MDCT can be used in this situation as the primary imaging method (Fig. 4). Routine use of MDCT in cases of high-energy trauma is associated with higher costs, radiation burden and numerous minor additional findings. Nevertheless, this procedure cannot be abandoned because there is a risk of omission of a clinically silent, curable life-threatening condition, such as aortic trauma [10].

### Examination technique

**Fig. 5:**
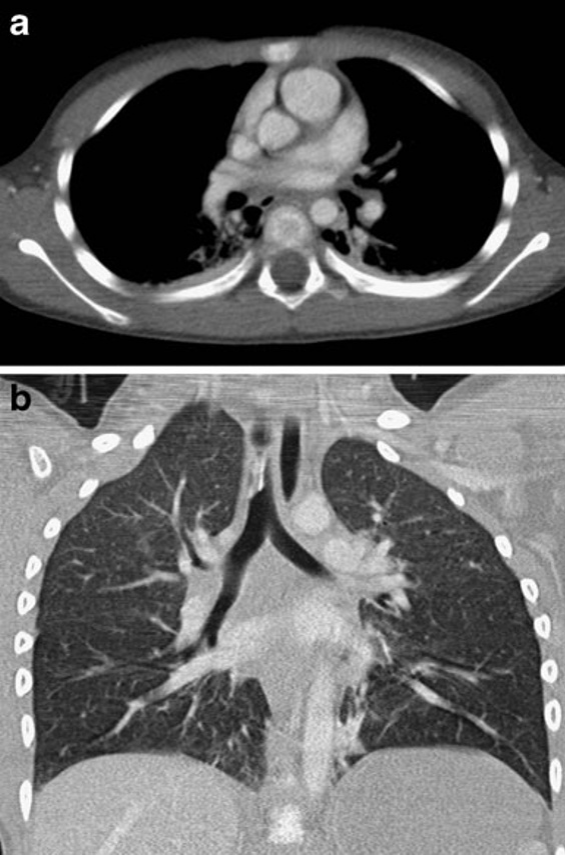
**a, b**Low-dose examination in a 6-year-old child. Significant dose reduction was achieved while maintaining an acceptable diagnostic quality (80 KV, 40 effective mAs, dose-length product 37 mGy*cm). **a**Axial slice, smooth reconstruction algorithm. **b**Coronal reformation, edge enhancement reconstruction algorithm

Examination protocols vary for different devices according to their technical parameters. Generally speaking, in order to minimise motion artifacts high examination speed should be used in traumas, and because of the need to perform reformations in other planes the highest resolution in the Z-axis should be used as well. The chest is an area with lower absorption of radiation and higher contrast between the single structures; therefore, in examination focused on the thorax a lower value of the exposure parameters can be used compared with the whole-body examination in which the abdominal area must be taken into account. It is suitable to apply dose modulation systems adjusting the voltage and current values according to a patient’s habitus and extent of absorption in the examined area [19]. Care concerning the radiation dose is especially important in children where we can reduce the burden from five to ten times compared with adults by using the appropriate exposure parameters. Dose reduction is achieved not only by using automatic dose modulation, but also by reduction of reference exposure values according to body weight. The kilovolt value can be reduced to 80–100, and the mAs value can be decreased to 30–80 (Fig. 5) [20]. If cardiac and thoracic aorta (especially a. ascendens) trauma is suspected, we can use ECG synchronisation to eliminate motion artifacts [21]. If it is possible with regard to the patient’s condition, the examination should always be performed with elevated upper limbs.

**Fig. 6:**
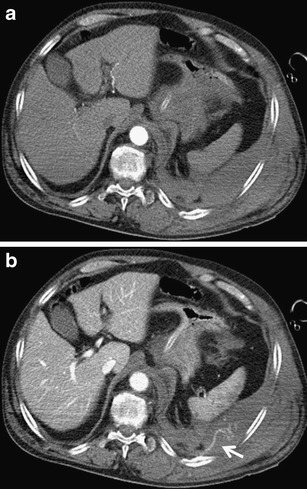
**a, b**Active bleeding into the extrapleural hematoma, importance of extended scanning delay after contrast injection. **a**Scanning delay 20 s: no evidence of contrast material extravasation. **b**Scanning delay 40 s: extravasation of contrast material from the intercostal artery (*arrow*)

Application of contrast media is essential for the assessment of vascular structures and parenchymal organs and for detection of active bleeding. Therefore, it is recommended to set a longer scanning delay (30–40 s.) compared with standard chest examinations (Fig. 6). [22, 23]. Examination of the chest trauma is usually performed without oral contrast agent application. A mall amount of contrast material can be used in case of suspected rupture of the esophagus, usually in addition to standard examination in unclear findings. In this case water-soluble iodinated contrast medium (usually 5–10 % solution) must be used, which does not cause complications and does not affect subsequent surgical therapy. Non-contrast examination in the primary diagnosis is not essential, but it can be used for follow-up examinations focused on the state of the lungs, bronchial tree or skeleton.

The slices reconstructed in three principal planes (axial, coronal and sagittal) or additional reconstructions in other planes and 3D images should be used for evaluation. To view the soft tissues, slices with a width of 3–5 mm reconstructed using a smooth resolution kernel are usually used. In lung parenchyma and bones, we use thinner slices (1–1.5 mm) reconstructed using a high-resolution kernel. A series of thin slices with a width of 0.6–1.5 mm with overlapping of about 1/4–1/3 (so-called secondary raw data) should be used for other 2D and 3D reformations. Using advanced software and powerful hardware 3D reformations are not time consuming. Their main contribution is a synoptic display of traumatic lesions of the skeleton, blood vessels or bronchial tree. In fact, they are not required for diagnosis alone, but may simplify assessment of anatomical relationships and communication with clinicians.

Imaging protocols for thoracic trauma are summarised in Tables 1and 2. Table 1shows protocols for different devices that are applicable in primary MDCT examinations. Table 2summarises modifications of standard protocol depending on clinical situation and purpose of the examination.

**Table 1:** Chest trauma protocols for contemporary MDCT devices

	10–16 rows	40–64 rows	>64 rows
Scanning parameters			
Voltage (kV)^a^	120	120	120
Current (eff. mAs)^a^	80–120	80–120	80–120
Rotation time (s)	0.5	0.33–0.5	0.27–0.5
Collimation (mm)	0.75–1.5	0.6–1.2	0.6
Pitch	1–1.5	0.9–1.5	0.9–1.5
Scan duration time (s)	5–10	2–5	1–3
Scan direction	Craniocaudal	Craniocaudal	Craniocaudal
Scan coverage	Lung apex to L1	Lung apex to L1	Lung apex to L1
Intravenous contrast injection			
Volume (ml)	80	80	80
Concentration (mgI/ml)	350–400	350–400	350–400
Flow rate (ml/s)	2.5–3	3–4	3–4
Iodine flux (mgI/ml)	1,000–1,200	1,200–1,400	1,200–1,600
Saline flush (ml; ml/s)	50; 2.5–3	50; 3–4	50; 3–4
Scan delay (s)^b^	Bolus triggering in ascending aorta (threshold 80 HU) + 15 or 30–40	Bolus triggering in ascending aorta (threshold 100 HU) + 15 or 30–40	Bolus triggering in ascending aorta (threshold 100 HU) + 15 or 30–40
Reconstructed images			
Soft tissue			
- Slice thickness (mm)	3–5	3–5	3–5
- Increment	3–5	3–5	3–5
- Recon. algorithm	Smooth	Smooth	Smooth
- Orientation	Axial, coronal, sagittal	Axial, coronal, sagittal	Axial, coronal, sagittal
Lungs, bones			
- Slice thickness (mm)	1–1.5	1–1.5	1–1.5
- Increment	1–1.5	1–1.5	1–1.5
- Recon. algorithm	High resolution	High resolution	High resolution
- Orientation	Axial, coronal, sagittal	Axial, coronal, sagittal	Axial, coronal, sagittal
Secondary raw data			
- Slice thickness (mm)	0.75–1.5	0.6–1.5	0.6–1.5
- Increment (mm)	0.5–1	0.4–1	0.4–1
- Recon. algorithm	Smooth, high resolution	Smooth, high resolution	Smooth, high resolution
- Orientation	Axial	Axial	Axial

Protocols are based on the experience of the authors with Siemens equipment; they are universally applicable in each thoracic trauma

^a^Using automatic dose modulation recommended

^b^Use of bolus triggering preferred

**Table 2:** Modification of a standard chest trauma MDCT protocol in different clinical situations

Chest MDCT as a part of the whole-body examination	Higher current value (adequate for examination of the abdomen)
	Contrast media volume 100 ml, flow rate 3–4 ml/s
	Trigger in descending aorta above the diaphragm (100 HU, delay 15 s) or scanning delay 30–40 s
Vascular trauma	Contrast media volume 80 ml, flow rate 4–5 ml/s
	Trigger in ascending aorta (80–100 HU, minimal possible delay—usually 4–5 s)
	Optionally ECG triggering (examination of ascending aorta or heart)
	Oblique reformated images in the plane of the aortic arch
	Optionally 3D reconstructions (volume rendering, MIP)
Bronchial tree trauma	Optionally 3D reconstructions (virtual endoscopy, MinIP)
Esophageal trauma	Optionally oral contrast agent (5–10 % solution of iodinated contrast material)
Follow-up examination focused on lungs, bones or bronchi	Optionally non-contrast
	Optionally low-dose examination (20–50 eff. mAs)
Children	80–100 kV^a^
	20–80 eff. mAs^a^
	Contrast media volume 1–2 ml/kg
	Flow rate dependent on needle size (22 G, 1.5 ml/s; 20 G, 2–3 ml/s; 18 G, 3–4 ml/s)

^a^According to body weight, in children with body weight more than 54 kg this is the recommended protocol for adults

## Chest wall injuries

Chest wall injuries are very common. Fractures reflect the intensity and direction of forces during trauma. However, the extent of a chest wall injury does not have to correlate with the injuries of the intrathoracic organs. This is evident especially in children and young adults with a flexible skeleton in whom severe visceral traumas may occur despite there being no fractures.

### Ribs

**Fig. 7:**
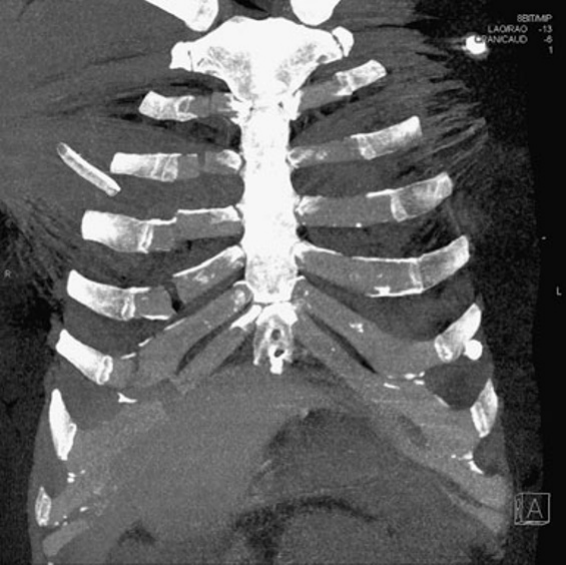
Multiple rib cartilage fractures. Coronal maximum intensity projection

Fractures of the ribs are the most common chest wall traumas. They affect approximately 50 % of patients and in fact are usually not clinically significant. However, their impact on adjacent intrathoracic and intraabdominal organs can be serious. In the case of a multiple fracture involving at least three consecutive ribs, a chest wall instability may occur that is associated with an impaired ventilation mechanism and increased risk of atelectasis (flail chest). The first three ribs are well protected by the collarbones, shoulder blades and muscles. Therefore, their fractures suggest a high-energy trauma. They may be associated with injury to the adjacent blood vessels or brachial plexus. In fractures of the lower three ribs, it is necessary to look for liver, spleen and kidney injuries. MDCT can determine the number of fractures, their location and degree of dislocation more accurately than X-rays. Contrary to X-rays, it is also able to detect fractures of the rib cartilages (Fig. 7) [3, 8].

### Scapula

**Fig. 8:**
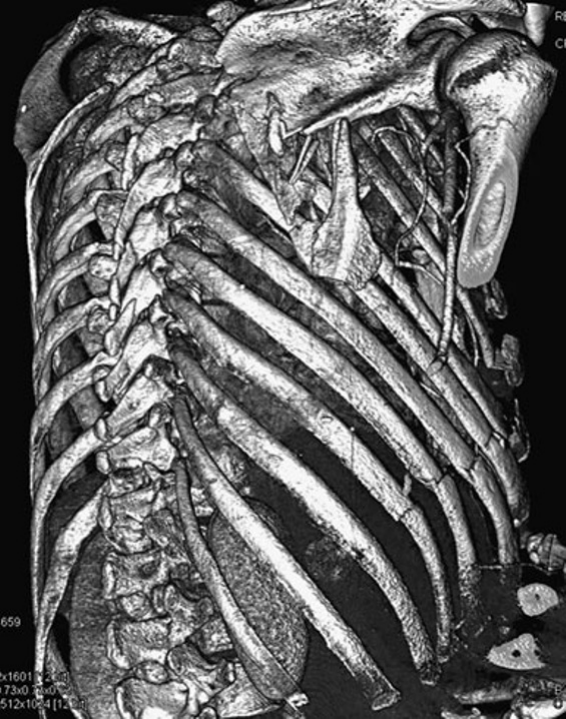
Synoptic view of the chest by using a volume-rendered image. Smash fracture of the right scapula and multiple rib fractures on the right

Fracture of the scapula belongs to the commonly overlooked injuries. It is usually caused by strong direct impact or the effect of large axial forces. In up to 40 % of cases, it is associated with pulmonary contusion, pneumothorax or hemothorax [24, 25]. The areas of the scapular body and neck are mostly affected (Fig. 8).

### Sternum

**Fig. 9:**
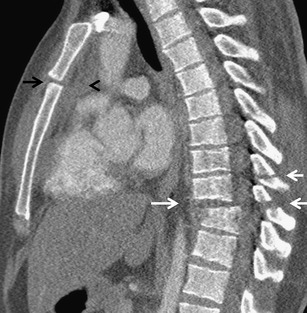
Unstable dislocated flexion-distraction fracture, type C1 according to the AO classification (*white arrows*), combined with dislocation in the sternal angle (*black arrow*) and retrosternal hematoma (*black arrowhead*)

Sternal fractures occur in 8–10 % of cases involving a blunt chest trauma. They are usually caused by direct impact on the anterior chest wall (typically impact with the steering wheel). They are often accompanied by retrosternal hematoma. Heart contusion is also present in 20–40 % of cases. Trauma of the mediastinal vessels can also be present. The retrosternal hematoma is usually separated from the aorta by a strip of fat. This symptom makes differentiation of the hematoma of aortal origin possible. A non-dislocated sternal fracture can be overlooked at the axial scans, and therefore sagittal reformations have the greatest importance for the diagnosis (Fig. 9) [3, 16, 26].

### Clavicle

Clavicle fractures are usually obvious from the clinical examination. MDCT is useful, especially in the diagnosis of sternoclavicular dislocation, which is caused most commonly by indirect mechanisms. Anterior dislocation is more common and clinically less severe. Posterior dislocations may be associated with major vascular traumas [27].

### Thoracic spine

Thoracic spine fractures represent 16–30 % of all spinal injuries. They are often difficult to detect using the X-ray examination because of superposition of other structures of the chest. The most common mechanism of injury includes flexion and axial forces. Rotating mechanisms are limited by the ribcage. Dislocations and unstable fractures, which occur in up to 50 % of cases associated with a neurological deficit, are relatively common in this area. Fractures are categorized into two groups: minor and major. Minor fractures (spinous, transverse and articular processes, interarticular part) alone are rarely associated with spinal instability or neurological deficits. According to the AO classification, major fractures are divided into (A) compression fractures, (B) distraction fractures and (C) multidirection injury with shear and/or translation. Assessment of fracture stability is crucial in deciding on the therapeutic approach. Unstable fractures are those that can lead to increasing deformity or increasing neurological deficits, and require surgical stabilisation. According to Denis’s three-column theory, fractures involving the middle column or two or more columns are regarded as unstable. CT is the first choice method for spinal fractures. Like in sternal fractures, sagittal reformations are very beneficial in this area (Fig. 9) [3, 28, 29].

### Extrapleural hematoma

**Fig. 10:**
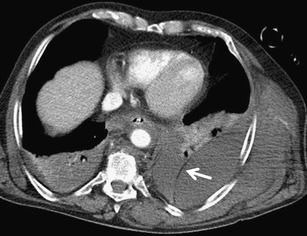
Combination of extrapleural hematoma and hemothorax. Lenticular-shaped extrapleural hematoma is surrounded by a thin strip of fat (*arrow*)

When extrapleural hematoma is caused by injury of the intercostal artery, it is a potentially life-threatening condition [30]. It occurs relatively rarely as a result of the chest wall injury or a complication of interventions (drainage, insertion of the central venous catheter) [31–33]. Blood accumulates between the parietal pleura and endothoracal fascia. The X-ray findings are often not specific. In the MDCT examination we can find blood collection separated from the lungs and pleural cavity by a thin strip of fat (Fig. 10) [33]. Larger hematomas have a biconvex shape [32]. Active bleeding from the intercostal artery may be seen in contrast material extravasation (Fig. 6).

## Injuries of the pleura

### Pneumothorax

**Fig. 11:**
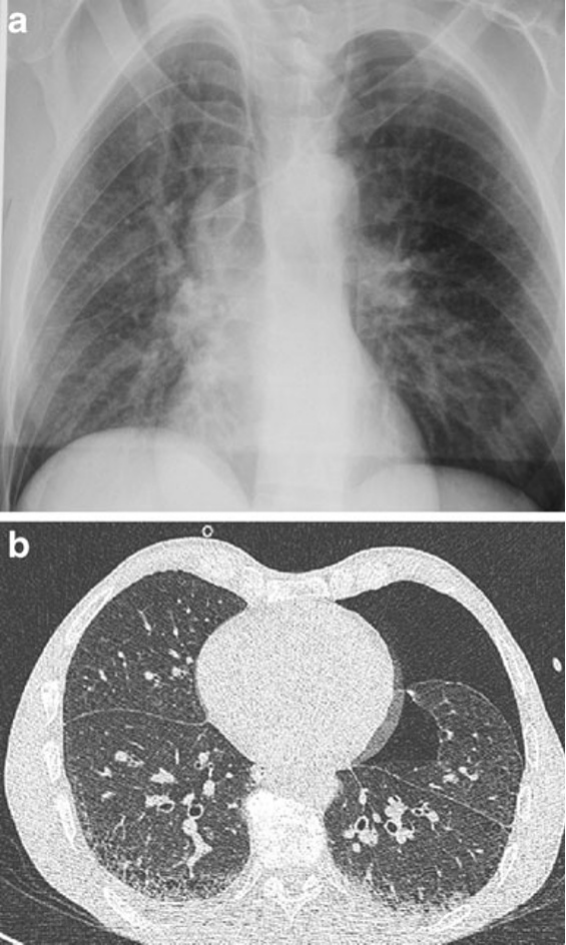
**a, b**Pneumothorax: discrepancy between the supine chest radiograph and MDCT. **a**Chest radiograph reveals slightly increased transparency of the left lung and thin linear translucency contouring the left border of the lower mediastinum. **b**MDCT shows significant pneumothorax in the ventral part of the left pleural cavity

**Fig. 12:**
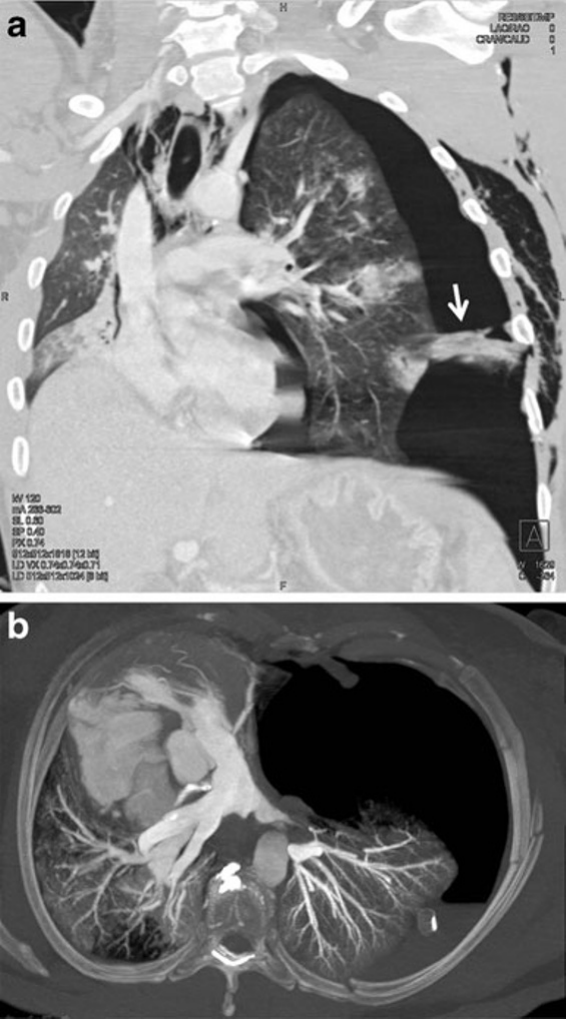
**a, b**Tension pneumothorax, indication for immediate chest drainage. **a**Increased volume of the left hemithorax, multiple lung contusions, and capture of the lung parenchyma among the fragments of the ribs (*arrow*). **b**Maximum intensity projection: mediastinal shift to the right, dislocation and compression of the heart, and compression of the left pumonary artery

Pneumothorax frequently accompanies blunt and penetrating chest injuries. In cases of blunt trauma, it is the second most common finding after rib fractures. It is caused not only by direct injury to the pleura, but also by a rupture of the alveoli or bronchi accompanied by increased pressure in the airways. It can also be a complication of medical procedures. On CT it is manifested as a collection of gas in the pleural cavity accumulating behind the ventral thoracic wall. In chest X-ray examinations performed in the supine position, it is often manifested very discretely, and in 30–55 % of cases it cannot be seen at all (Fig. 11) [34, 35]. The clinical significance of pneumothorax depends not only on its size at the time of initial examination, but also on its development over time and on the overall condition of the patient. In a ventilated patient, a small overlooked pneumothorax can grow rapidly and cause a hemodynamic and ventilation instability [35]. Tension pneumothorax is a serious, life-threatening condition requiring immediate drainage. It results in an increase of intrathoracic pressure on the affected side with a subsequent compression of the mediastinal structures and reduction of cardiac diastolic filling [36]. It is often clinically diagnosed before MDCT. Its basic symptoms include an increase of the volume of the affected hemithorax, shift of the mediastinum to the healthy side, and compression of the mediastinal vessels (mainly veins), heart and depression of the diaphragmatic arch (Fig. 12) [16].

### Hemothorax

**Fig. 13:**
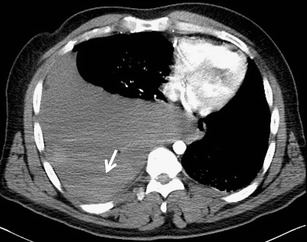
Large layered hemothorax. Hyperdense clots (50 HU) in the dorsal part of the right pleural cavity (*arrow*). Mediastinal shift to the left side, compression of the right atrium

Bleeding into the pleural cavity is most commonly caused by laceration of the pulmonary parenchyma and pleural injury. In these cases, bleeding is usually slowly progressive and limited. In case of injury to an artery (usually intercostal), bleeding progresses rapidly and requires surgical therapy. Less extensive bleeding does not have to be detected on the X-ray examination performed in the supine position. MDCT is an unrivalled method for the diagnosis of pleural fluid. Blood has a higher density than water. Its value depends on the degree of coagulation. The density of liquid blood is between 30 and 50 HU, and the density of blood clots is 50–90 HU. Hematomas can sometimes have a layered structure (so-called hematocrit sign) (Fig. 13). In case of active bleeding from the injured artery, we can demonstrate leakage of the contrast substance into the hematoma [3, 16]

## Lung parenchymal injuries

Injury to the lung parenchyma includes pulmonary contusion, laceration, torsion and herniation. These conditions can be complicated by atelectasis, aspiration pneumonia or acute respiratory distress syndrome (ARDS) [9]. Lung trauma is seldom a reason for surgical treatment. This is indicated in cases of injury of the major blood vessels, signs of active bleeding, large hematomas or hemodynamic instability in the context of pulmonary involvement.

### Pulmonary contusion

**Fig. 14:**
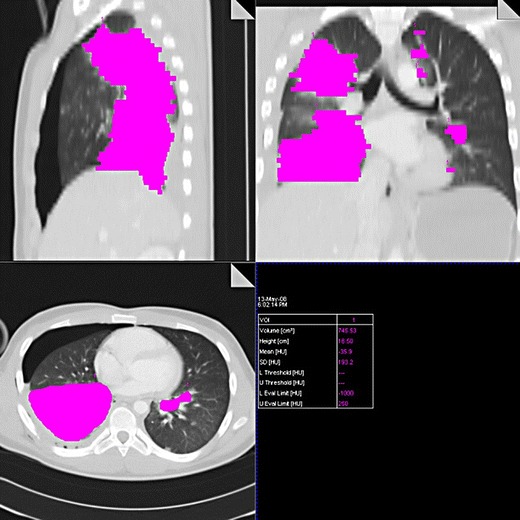
Lung contusion volumetry using semiautomatic software volume (Siemens, Forchheim, Germany). Highlighted contusions occupy 26 % of the total lung volume. The finding indicated artificial ventilation

**Fig. 15:**
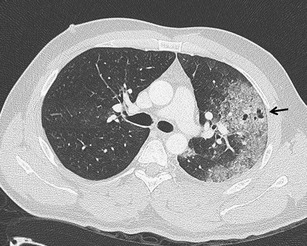
Lung contusion with small lacerations (arrow), typical peripheral localisation

**Fig. 16:**
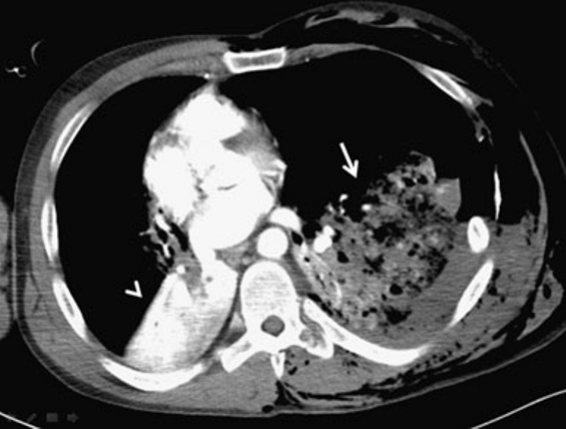
Comparison between contusion and atelectasis. Contusion (*arrow*) is an inhomogenous, ill-demarcated, relatively hypodense parenchymal condensation with preservation of air in the bronchi (air bronchogram). Atelectasis (*arrowhead*) appears like homogenously enhancing well-demarcated parenchymal condensation without an air bronchogram

**Fig. 17:**
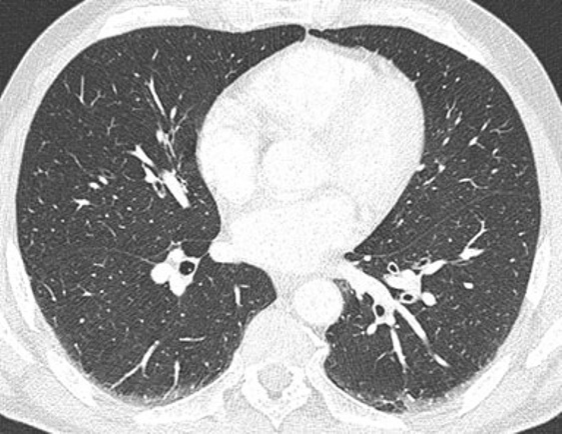
Dependent density: symetrical bilateral sickle-like condensations in dependent parts of both lungs

Pulmonary contusion is the most common trauma of the lung parenchyma with an incidence between 30 % and 70 %. It leads to hemorrhage, edema and secondary inflammatory infiltration of the pulmonary interstitium and alveoli [16]. It can be detected on the X-ray image with up to a 6-h delay. On the other hand, CT is very sensitive to contusion changes, and it can detect them earlier and more accurately and allows their quantification, which is important in the determination of further treatment. In contusions exceeding 20–30 % of the total lung volume, mechanical ventilation is indicated to prevent the development of complications, especially acute respiratory distress syndrome [37, 38]. The volume of the affected parenchyma can be estimated (each segment represents about 5 % of the total lung volume) or exactly measured using dedicated volumetry software. The second option is more time-consuming (Fig. 14). If the CT scan is performed immediately after the trauma, further progression of the findings must be taken into account. Contusion achieves its maximum in the 24–48 h followed by absorption, which takes 1–2 weeks [3, 5]. The CT findings depend on the severity of parenchymal affection. In cases of interstitial involvement, areas of ground-glass opacity occur. More serious affection is accompanied by inhomogeneous alveolar condensation. Simultaneously, parenchymal lacerations can occur (Fig. 15). The peripheral areas of the lung are affected, and the distribution does not respect boundaries of the lobes and segments [9]. Differentiation of the contusion is difficult in the field of atelectasis, compression of the parenchyma with hemothorax or aspiration pneumonia (Fig. 16). The dependent density (the sickle-shaped parenchymal condensations in the dependent lung regions) should not be considered as contusions (Fig. 17) [39].

### Lung laceration

**Fig. 18:**
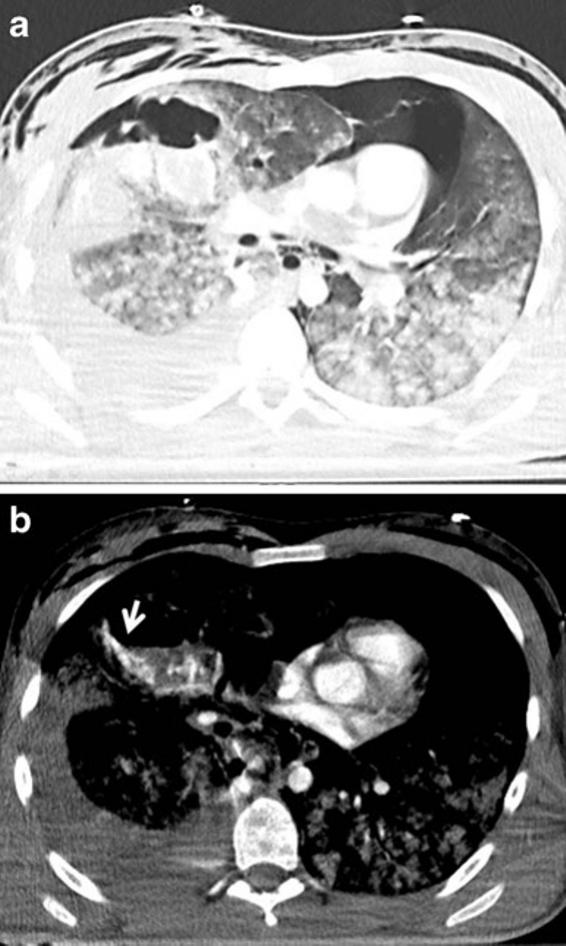
**a, b**Extensive lung laceration, large bilateral contusions. **a**Cavities with air-fluid levels in the right upper lobe, right-sided pneumothorax and fluidothorax, bilateral subcutaneous emphysema, pneumomediastinum, mediastinal shift to the left. **b**Extravasation of the contrast material into the cavity due to active bleeding; the finding was an indication for surgery

Laceration occurs in penetrating injuries as a result of direct tearing of the parenchyma in blunt traumas as a result of shearing forces. Laceration was considered a rare finding before the era of CT. At present we can find it relatively commonly because of the broad use of CT in traumatised patients. X-ray examination is usually negative because of superposition of simultaneously occurring contusion changes. If a laceration reaches the pleural cavity, it can cause pneumothorax or hemothorax. Laceration closed in the parenchyma has the appearance of a thin-walled, spherical, oval or tubular cavity containing air, blood or their combination (Fig. 18). There are usually contusion changes around it. When they are absorbed the laceration, which does not contain air, can mimic a pulmonary node [9, 16].

### Lung torsion

Lung torsion is a rare, life-threatening complication of pulmonary surgery or trauma. In rare cases it can occur spontaneously. It affects the whole lung or a lobe [40]. On CT the torsion is manifested as an abnormal position of the lung or its lobe with condensation and obstruction of the airways or distortion and abnormal position of the bronchi and blood vessels [41].

### Lung herniation

Like torsion, lung herniation is a rare complication of chest injuries. It occurs at the site of an inherited or acquired defect of the chest wall with a significant increase in intrathoracic pressure. The acquired chest wall defects can be caused by multiple fractures of the ribs or by sterno- and costoclavicular dislocation. If parenchymal tearing occurs at the same time, the herniation may be associated with pneumothorax and hemothorax [16, 42].

## Injuries of trachea and bronchi

**Fig. 19:**
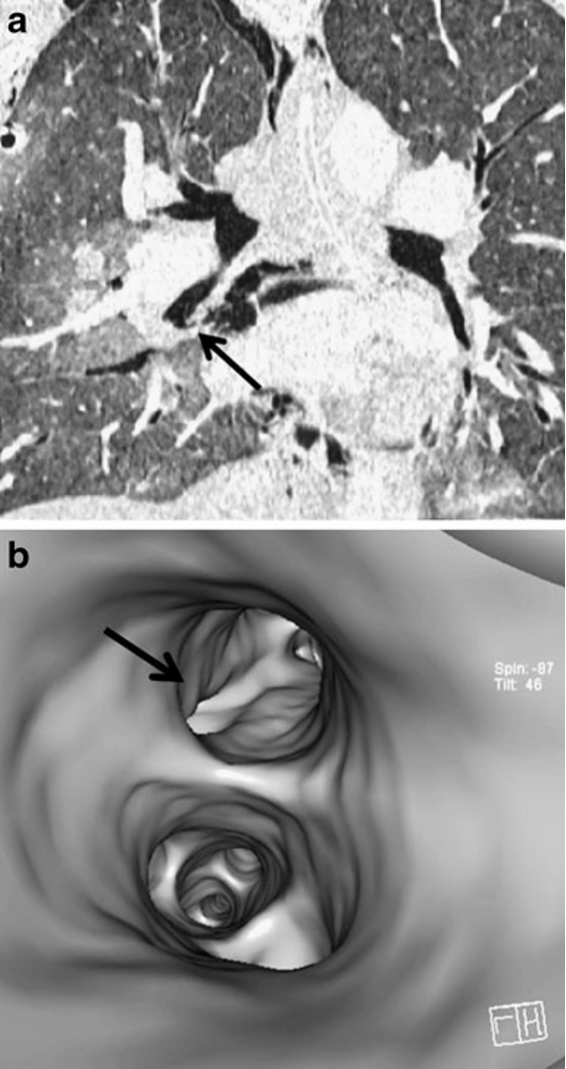
**a, b**Tiny tear of the right middle lobar bronchus, contribution of virtual bronchoscopy. **a**Multiplanar reformation—the tear is poorly visible (*arrow*). **b**Virtual bronchoscopy: clear visualization of the tear (*arrow*)

**Fig. 20:**
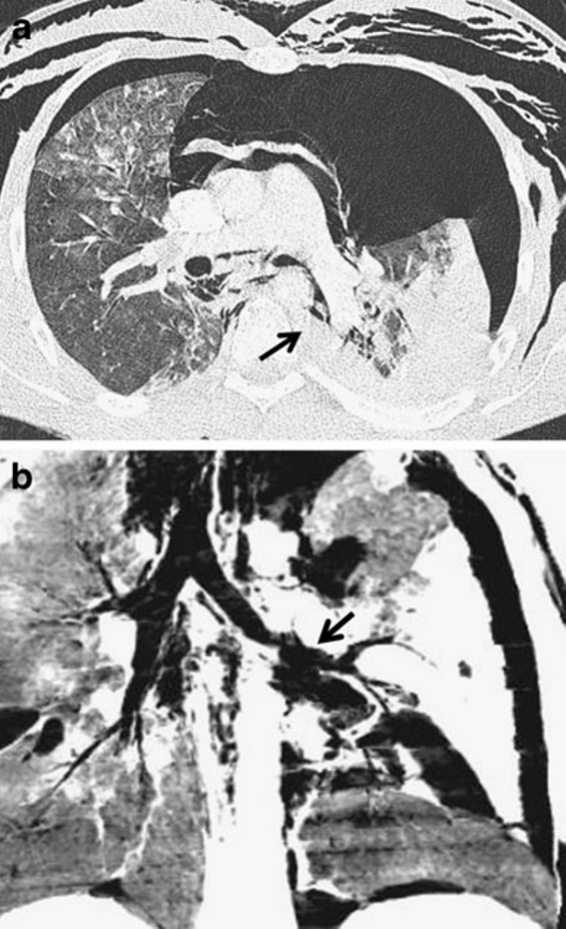
**a, b**Rupture of the left main bronchus (*arrow*). **a**Discontinuity of the bronchus, fallen lung sign, pneumomediastinum, bilateral pneumothorax and subcutaneous emphysema. **b**Coronal minimum intensity projection shows leakage of air through a tear

Laceration of the trachea and bronchi is caused by two mechanisms—by increasing the intraluminal pressure with a closed vocal slot due to a sudden compression of the chest or by compression of the main bronchi against the spine. The neck part of the trachea can also be injured by a direct impact. The most common location of lacerations in the trachea is 2 cm above the carina and in the main bronchi (more in the right) at a distance of 2.5 cm from the carina [3, 22]. A tracheobronchial trauma is often difficult to find. In addition to thin slices in the axial plane and multiplanar reformations, the 3D visualisation of the bronchial tree (using virtual bronchoscopy or the volume-rendering technique) can be helpful for detection of the rupture (Fig. 19) [43]. A defect in the wall of the airways with leakage of air into the surrounding area is a direct sign of the airway trauma. There are other relatively specific findings such as a collapsed lung located in the most dependent part of the hemithorax (fallen lung sign) and persistence of pneumothorax following the insertion of the intrathoracic drain (Fig. 20). Other symptoms, such as pneumomediastinum, subcutaneous emphysema in the neck and chest, pneumothorax and hemothorax, are nonspecific and more commonly have other causes [44].

## Esophageal rupture

Rupture of the esophagus as a part of a blunt or penetrating chest trauma is rare. Most often, it occurs in the cervical and upper mediastinal part. It is usually caused by bone fragments from the injury of adjacent sections of the spine. The CT findings include thickening of the esophageal wall, pneumomediastinum and paraesophageal fluid collections and fluidothorax (more often in the left) [45, 46]. If an oral contrast agent is administered before CT, its leakage through the esophageal wall defect can be demonstrated [22].

## Diaphragmatic rupture

**Fig. 21:**
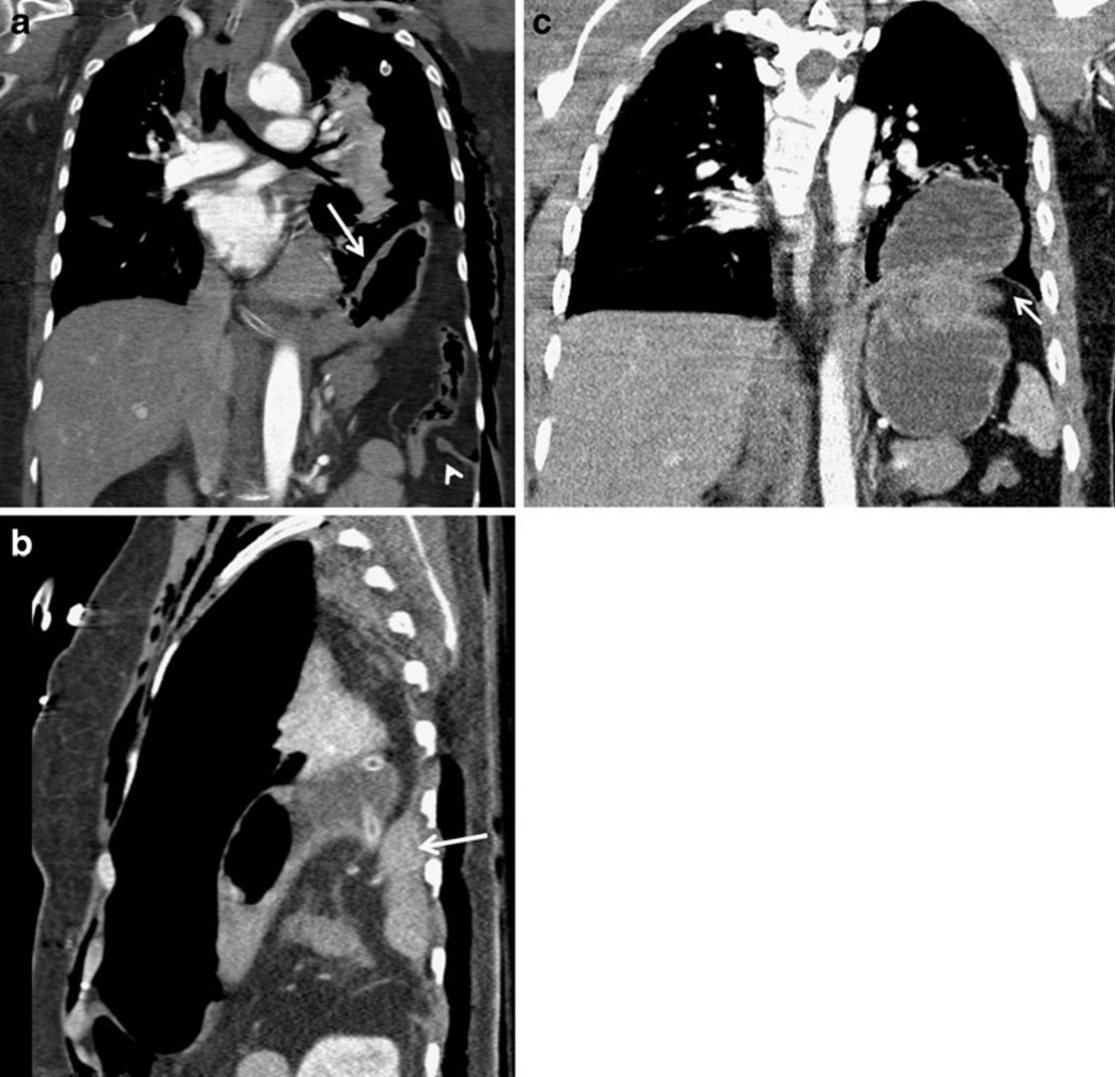
**a, b, c**Signs of rupture of the diaphragm. **a**Herniation of abdominal organs into the left hemithorax (*arrow*), club-shaped edge of the ruptured diaphragm (*arrowhead*). **b**Contact of herniated viscera with the most dependent part of the chest wall: dependent viscera sign (arrow). **c**Strangulation of herniated viscera in the defect of the diaphragm: collar sign (*arrow*)

Diaphragmatic rupture occurs in 0.8–7 % of patients hospitalised with a blunt trauma [47]. It is a frequently overlooked injury but it is clinically very serious. It occurs more often on the left (2/3–3/4 of cases), probably because the right half of the diaphragm is protected by the liver. Laceration in the right side is more difficult to detect. Sensitivity of the initial X-ray examination is low, 17 % and 27–60 % on the right and left side, respectively [48]. Compared to this, MDCT reaches a sensitivity of 50–83 % and 78–100 % in the right and left side, respectively. Rarely, herniation to the pericardium or through the hiatus esophagus can occur [8]. Evaluation of multiplanar reformations in the coronal and sagittal plane is very important for making the diagnosis [49–53]. Diaphragmatic rupture is associated with a herniation of the abdominal organs, which can arise at the time of injury or later. The CT signs of a diaphragmatic rupture include discontinuity of the diaphragm with thickening at the edge of the defect, herniation of the abdominal organs into the chest, strangulation of the organs at their passage through the diaphragmatic defect (collar sign) and location of herniated abdominal organs in the dorsal part of the thorax where they are in contact with the chest wall (dependent viscera sign) (Fig. 21) [50]. Diaphragmatic injuries are often associated with injuries of the spleen, liver, lungs and rib fractures [54].

## Trauma of large vessels

Trauma of the large chest vessels represents about 5 % of all vascular traumas, but its incidence is probably underestimated. Although relatively infrequent, it is the second most common cause of death after head injuries [55, 56]. The aorta is the most commonly affected vessel. Rarely, we can see injuries of the aortic branches, internal thoracic artery or major mediastinal veins. In the case of a blunt trauma, there are three traumatic mechanisms leading to the trauma—deceleration, torsion and increased intraluminal pressure. Findings on the X-ray examination are usually nonspecific and do not allow to differentiate trauma of the large vessels from other less serious conditions with similar X-ray findings, as described above. [5, 12]. If this type of trauma is suspected, MDCT is currently the first-choice method. Its sensitivity and specificity are almost 100 %. Angiography is indicated only in cases of doubt and for navigation of the interventional procedures [57].

### Aortic trauma

**Fig. 22:**
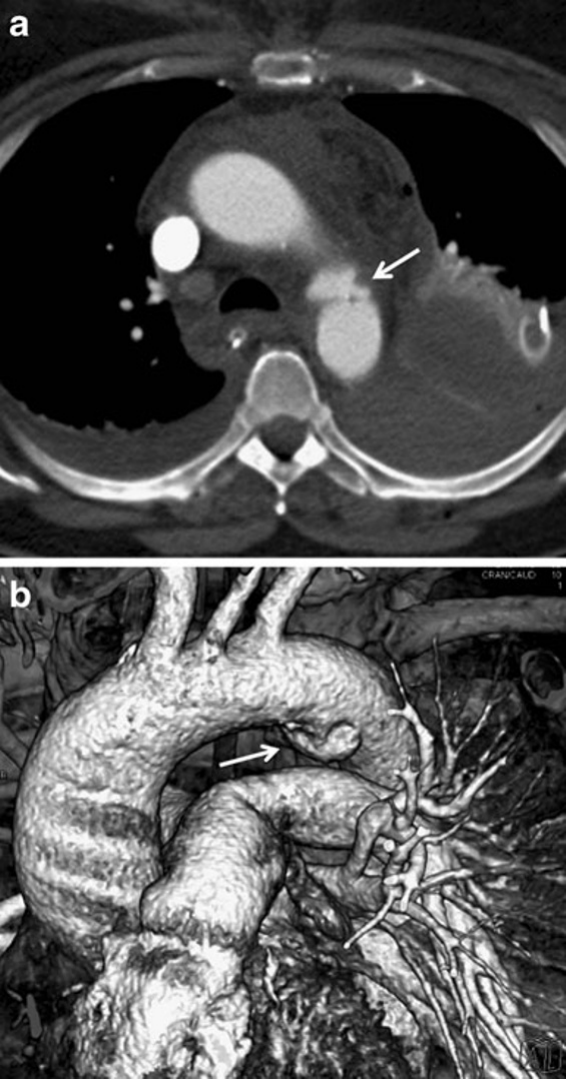
**a, b**Aortic isthmus laceration. **a**Pseudoaneurysm in a typical location (*arrow*), mediastinal hematoma, bilateral hemothorax. **b**Volume-rendered image: 3D view of the pseudoaneurysm (*arrow*)

**Fig. 23:**
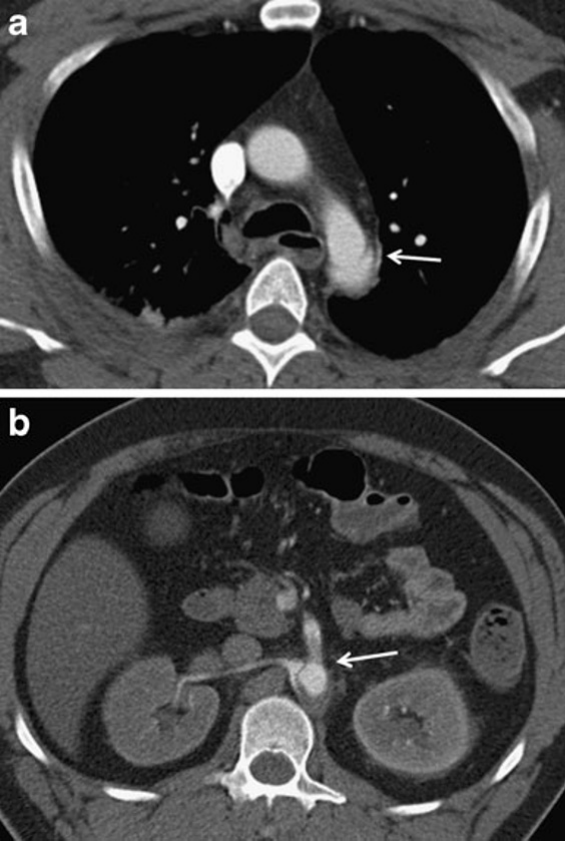
**a, b**Traumatic dissection of the descending aorta. **a**Proximal border at the level of the isthmus. **b**Involvement of the abdominal part

**Fig. 24:**
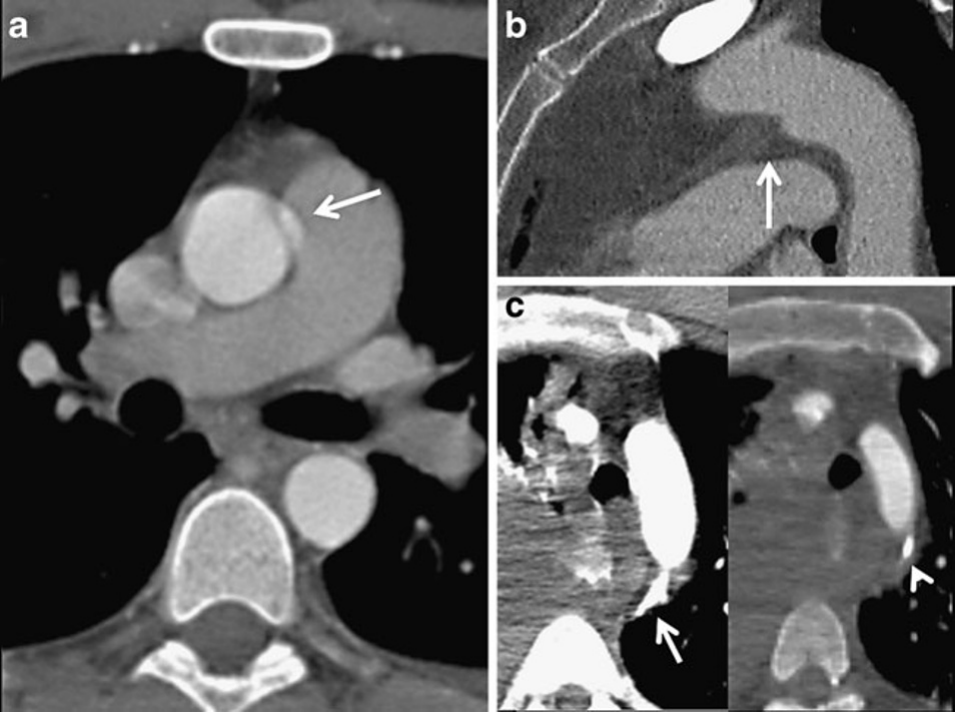
**a, b, c**Pitfalls in diagnostics of aortic trauma. **a**Motion artifact mimicking a tear of the ascending aorta. **b**Ductus diverticulum: smooth shape, no mediastinal hematoma, connection to the falciform ligament (*arrow*). **c**Supreme intercostal vein (*arrow*). Note the different density after correcting the window setting (*arrowhead*)

Mortality of untreated aortic trauma exceeds 95 % [58]; 80–90 % of patients die at the place of accident. Those who come to the hospital have a 70 % chance of survival if the trauma is quickly diagnosed and treated [59, 60]. Treatment is either surgical or radiointerventional (endovascular stent grafting). The most common site of injury (approximately 90 %) is the isthmus, which represents the transition between the relatively mobile arch and fixed descending part. Other less frequent localisations of trauma include the ascending aorta above the aortic valve, branching of the truncus brachiocephalicus and the distal segment of the descending aorta. Impairment of the wall spreads from the intima to adventitia [3, 61, 62]. In patients who come to the hospital, we can more often find partial lesions. A complete wall rupture (transsection) usually results in immediate death. Direct signs of aortic trauma are pseudoaneurysm (Fig. 22), direct extravasation of contrast medium into the mediastinum, intimal flap (Fig. 23), irregular contour, mural thrombus or pseudocoarctation [5, 12]. Indirect and less specific signs of aortic trauma include periaortic hematoma, hemothorax and hemopericardium. Periaortic hematoma may come from small blood vessels in the affected area or from the vasa vasorum and may not be a sign of aortic wall injury. Problems with interpretation of findings may arise in case of ulcerated atherosclerotic plaques or congenital anomalies such as the ductus diverticulum or supreme intercostals vein. In these cases, the aorta is externally well demarcated and without periaortic hematoma. The edge of the ductus diverticulum is smooth and regular [63]. In some cases we can identify the ligamentum arteriosum, which is linked to it. The supreme intercostal vein flows into the brachiocephalic vein and may run along the left side of the aortic arch. On the ascending aorta above the valve, motion artifacts can often be seen (Fig. 24). In case of diagnostic uncertainty, it is possible to add a targeted examination with ECG synchronisation.

### Injuries of other large vessels

**Fig. 25:**
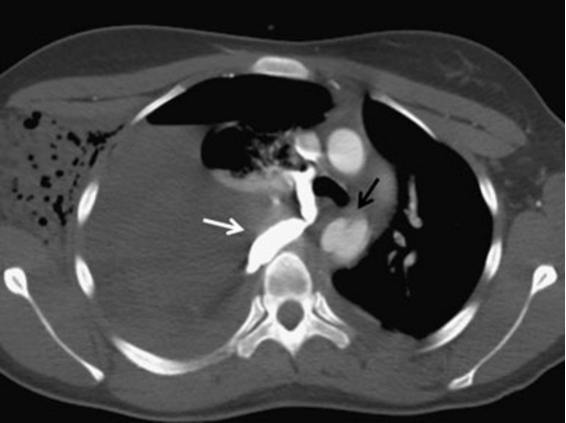
Rupture of the azygos vein with contrast material leakage into the large hemothorax (*white arrow*) combined with aortic pseudoaneurysm (*black arrow*)

Injuries of other blood vessels, such as the internal thoracic artery, branches of the aortic arch, pulmonary artery or mediastinal veins, are less common than aortic trauma, but their severity is similar. In case of bleeding from the internal thoracic artery, a hematoma occurs in the anterior mediastinum, and it may compress the right ventricle [64]. Injury of the branches of the aortic arch occurs most frequently in the traction mechanism in case of abnormal extension of the neck or pulling of the arm. It is manifested by occlusion, dissection of the affected artery or extravasation of contrast material into the hematoma, which is typically located in the upper part of the mediastinum. Involvement of the common carotid artery can cause stroke [8]. Rare injuries of the venous structures are manifested as hematoma with extravasation of contrast medium (Fig. 25). The mechanism is similar to injuries of the arteries. Injury of the vena cava superior can spread to the right atrium as well [65, 66].

## Heart and pericardium injuries

**Fig. 26:**
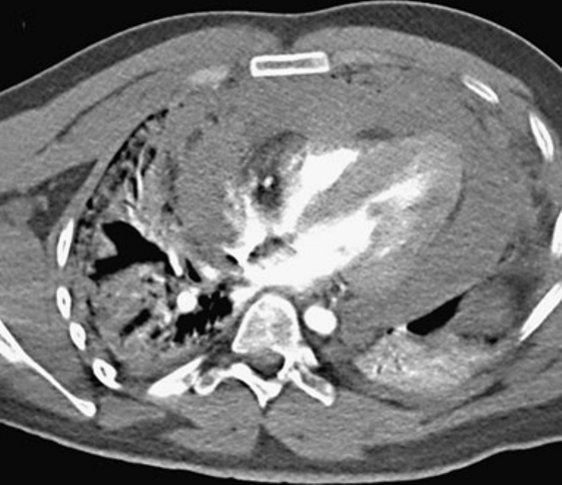
Hemopericardium with heart tamponade: compression of all heart parts

Contusion is the most common heart injury affecting usually the right ventricle. It is generally radiologically silent and causes arrhythmia or other electrocardiographic changes. Because of its very high mortality, cardiac rupture is rarely diagnosed by imaging methods [67]. It can be manifested as a hemopericardium and extravasation of contrast material from the injured cardiac cavity [68]. Rarely, due to a coronary artery injury, myocardial infarction can occur [69]. Diagnosis of hemopericardium using X-ray examination is extremely problematic. The finding can be negative even in cases of cardiac tamponade. In contrast, the CT examination as well as ultrasound is a highly sensitive method. Cardiac tamponade is manifested by flattening and a reduction of the cardiac parts (Fig. 26) [70]. More rarely, we can see pneumopericardium, which, if large, may also result in cardiac tamponade [71]. Herniation of the heart is a serious complication of traumatic rupture of the pericardium [72].

## Conclusion

Imaging methods are an integral part of the diagnostic algorithm in chest injuries. MDCT is its main component. It shows traumatic changes quickly, accurately and clearly, and allows their classification. Therefore, a traumatic radiologist becomes a significant member of the team making decisions about the therapeutic process.
